# Acceptance and Impact of Millet-Based Mid-Day Meal on the Nutritional Status of Adolescent School Going Children in a Peri Urban Region of Karnataka State in India

**DOI:** 10.3390/nu11092077

**Published:** 2019-09-03

**Authors:** Seetha Anitha, Joanna Kane-Potaka, Takuji W. Tsusaka, Deepti Tripathi, Shweta Upadhyay, Ajay Kavishwar, Ashok Jalagam, Nidhi Sharma, Swamikannu Nedumaran

**Affiliations:** 1 International Crops Research Institute for the Semi-Arid Tropics (ICRISAT), Hyderabad 502324, India; 2 ICRISAT, Lilongwe 1096, Malawi; 3 Organization for Advanced and Integrated Research, Kobe University, Kobe 650-0047, Japan; 4 Akshaya Patra Foundation, Bangalore, Karnataka 560022, India; 5 United Nations Children’s Fund (UNICEF), Lilongwe 30375, Malawi

**Keywords:** school feeding program, mid-day meal, millets, sensory evaluation, undernutrition

## Abstract

The study assessed the potential for use of millets in mid-day school meal programs for better nutritional outcomes of children in a peri-urban region of Karnataka, India, where children conventionally consumed a fortified rice-based mid-day meal. For a three-month period, millet-based mid-day meals were fed to 1500 adolescent children at two schools, of which 136 were studied as the intervention group and were compared with 107 other children in two other schools that did not receive the intervention. The intervention design was equivalent to the parallel group, two-arm, superiority trial with a 1:1 allocation ratio. The end line allocation ratio was 1.27:1 due to attrition. It was found that there was statistically significant improvement in stunting (*p* = 0.000) and the body mass index (*p* = 0.003) in the intervention group and not in the control group (*p* = 0.351 and *p* = 0.511, respectively). The sensory evaluation revealed that all the millet-based menu items had high acceptability, with the highest scores for the following three items: finger millet *idli*, a steam cooked fermented savory cake; little and pearl millet *bisi belle bath*, a millet-lentil hot meal; and *upma*, a pearl and little millet-vegetable meal. These results suggest significant potential for millets to replace or supplement rice in school feeding programs for improved nutritional outcomes of children.

## 1. Introduction

India has one of the world’s highest demographics of children suffering from various types of malnutrition, which is double that in Sub-Saharan Africa [1]. A total of 44% of children under the age of five are underweight, while 72% of infants have anaemia [2]. In particular, the rate of undernutrition from lack of micronutrients, especially iron deficiency anaemia, is high in India. According to India’s National Family Health Survey [3], more than half the women (55%) have iron deficiency anaemia. In addition, underweight, wasting, and stunting are also prevalent in children under five (36%, 21% and 38%, respectively) [3]. These figures hint at serious long-term consequences for human capital development and the productivity potential of the nation. More importantly, they also imply the denial of basic human rights of children, such as access to food.

India has one of the fastest growing youth populations in the world. The distribution of 10–19 year olds stands at 19.5% of the total population [3]. Adolescence is a critical stage in human life when physical growth is high; so is the nutrient requirement. 

India’s school feeding programs (SFP) known as the mid-day meal (MDM) programs are aimed at improving nutrition intake, and enrolment and retention at school [4]. SFPs have been seen as both a social safety net for vulnerable segments of the population and an educational intervention to incentivize children to go to school, where their learning ability in the classroom is improved by reduction in hunger. Under MDM programs, children at all public and government-assisted primary schools are provided with a prepared mid-day meal containing a minimum of 450 kilocalories and 12 grams of protein per school day for primary school students and 700 kilocalories and 20 grams of protein for grade 6–8 students for a minimum of 200 days a year [5,6]. Several studies have demonstrated the positive impacts of mid-day meals [7,8]. The MDM comprises a freshly cooked meal containing food grains, mainly rice (*Oryza sativa* L.), legumes and vegetables. In general, the MDM in India provides cereal on a regular basis in adequate quantities, while pulses and vegetables are inadequate due to their high prices [9]. In other words, the MDM’s major ingredient is a cereal, particularly rice and wheat. Given the extent of undernutrition in children and adolescents, there is scope to improve the nutrient content of MDMs by providing more nutritious food using crops that are locally available and traditionally used. 

The Global Hunger Index (GHI), 2018 report indicates that the level of hunger and undernutrition is serious and it is 20.9% worldwide [10]. This underlines the need for sustainable crop substitutes to mitigate hunger and to improve farmers’ incomes globally. The role of millets in achieving sustainable means of nutritional security cannot be ignored as they are more climate change compliant crops than cereals, such as wheat (*Triticum aestivum* L.) and rice, given the marginal conditions under which they are grown and their high nutritional value [11]. The vital micronutrients and protein in millet grains are considered to be equal to or superior to those in wheat, rice, maize (*Zea mays* L.) and sorghum (*Sorghum bicolor* L.) grains [12]. In particular, the mineral content in millets is several folds higher than that in wheat and rice [12] and can complement legumes for the amino acid profile, especially methionine. On the other hand, as many other cereals and legumes, millets contain antinutrients, such as phytic acid, which inhibits the absorption of minerals, leading to iron and zinc deficiencies [12]. Hence, dephytinization practices must be imparted when promoting recipes containing millets, especially in resource poor environments. Various processing methods and in-home food preparation techniques can alter the phytic acid content and improve the bioavailability of micronutrients. Simply cooking the millet ingredients can reduce the phytic acid to some extent. Some studies also show that pearl millet exhibits goitrogenic effects on millet consuming population through iodine deficiency [13]. Since there is not enough evidence, such as data on dietary iodine intake by the studied population, further long-term studies are needed to assess the amounts of millets, varieties of pearl millet, and the extent of their influence on goiter. In short, to maximize the benefits of millet-based food, preparation methods need to be taken into account in designing nutrition intervention programs utilizing millets. While millets are traditional grains of many countries including India, they are also recognized as “Smart Food” since they are both climate stress resistant and naturally rich in micronutrients. In other words, they are “good for you, the planet and the farmer” [14]. Considering these multiple benefits, millets are arguably the best fit to fill the gap in agricultural food systems [11]. 

Over the decades, millets have become less and less common in India in terms of both production and consumption [15], partly because of the limited knowledge on their use in preparing various foods. At present, finger millet is used in Karnataka mostly to make finger millet rice ball, and pearl millet is used in baking roti together with wheat. To meet the need for variety and taste for children, it is important to use these grains in other forms of food in a culturally acceptable way. 

Lately, these nutritional and drought-resistant properties of millets have drawn the attention of research agencies and product developers globally, increasing the focus on improving millet varieties and enhancing their use in modern processed food products. The current study was conducted to: (a) analyse the impact of regular consumption of millet-based recipe formulations on children’s nutritional outcomes; (b) assess the acceptance of millet-based recipe formulations among children especially as a staple; (c) evaluate the suitability of these formulations to be adopted in MDM.

## 2. Materials and Methods

Akshaya Patra operates the largest school feeding program in India supported by the Government (https://karnataka.akshayapatra.org/), feeding over 1.7 million children across the nation. After briefing the government, the millet-based feeding program to the selected Akshaya Patra beneficiary school children was approved in writing by the Government of Karnataka, Department of Education and Department of Agriculture. Along with this approval, meetings were held with school principal and parents/guardians of children to discuss the purpose, procedures and risks involved in the study. Informed written consent was obtained from the head of the schools and parents/guardians of children involved in the study based on written and verbal information provided before the program commenced.

Site: The study was conducted in four public schools in a peri-urban region of Bengaluru (Thathaguni, Kagallipura, Allahali and Chensandra villages), Karnataka state of India, where children came from low and middle income families.

**Figure 1:**
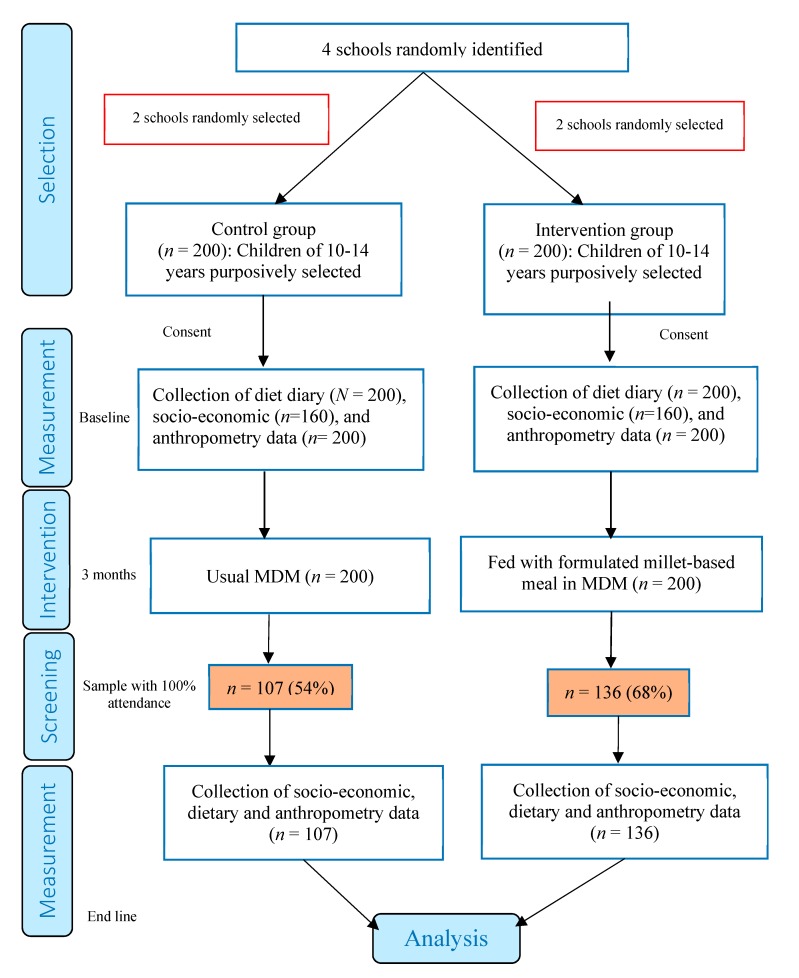
A flow diagram for the study.

Sampling framework: Figure 1 provides the flow chart of the study including the sampling framework. The study chose 400 children between the ages of 10 and 14 from four schools who expressed interest in participating in the study for the three-month period from July to September 2018. Children with no history of food allergies and who attended school regularly were purposively selected. All the sample children consumed rice in MDMs before the intervention. The intervention was provided school-wise in light of feasibility of administration. Of the 400 children, 200 from two randomly selected schools were chosen as the control group, and the other 200 (from the other two schools) fell into the intervention group. The initial consent was obtained from all the 400 respondents and the baseline assessment was conducted with all of them. Nonetheless, full attendance to school throughout the study period was a requirement in order for the respondents to qualify for inclusion into the end line assessment. As a result, 32% of the children in the intervention group and 46% of them in the control group were excluded from the end line assessment as they did not attend school regularly. The 400 participating children were given Smart Food identification cards to distinguish them from other children consuming the same food but not part of the study. In the two intervention schools, around 1500 children were fed with the same millet-based meals.

Consent obtained: Before starting the program, formal written consent was obtained from both parents and the school management. Approval was also obtained from the State Government to introduce millet-based food during the MDM program for our research.

Data collection: A survey instrument was designed, which was reviewed by a panel of nutrition experts to examine the face validity, readability and clarity of wording and instructions. The instrument had two parts: household dietary assessment to record family dietary intake [16] and a diet diary to record the children’s dietary intake for three days, which was for normal week days, including no special or fasting day nor weekends. These instruments were used to collect dietary data before starting the intervention. The details follow.

### 2.1. Household Dietary Assessment

Household dietary assessment was conducted in the households of randomly selected 10% of the sample children. The one-day weighment method was followed to estimate nutrient intake [17,18,19]. The dietary patterns were recorded along with each recipe that was prepared during the entire day of the visit. All the ingredients used in each recipe were weighed using a grocer’s scale, and the quantity used to cook for the household members was documented. The numbers, thickness and dimensions of the food items, such as steam cooked rice cake and wheat rotis, were recorded. The quantity of food items served using various serving spoons was measured and recorded to estimate food intake. The food intake data was then used to calculate the nutrient intake using the DietSoft (India, www.DietSoft.in/) online software [19] based on the Indian Food Composition Table [20].

### 2.2. Individual Dietary Assessment

The sample children were trained to fill out a diet diary for three week days of what they ate from the time they woke up in the morning to the last item consumed before going to bed at night. The timing of food consumption was recorded. The diet diary was used to provide the estimated amount of food consumed, instead of weighed quantities consumed. For each food item, the amount consumed was recorded in household measurement (spoons, ladles or cups), numbers or dimensions, whichever was appropriate. The containers most commonly used in the communities were large, medium and small serving spoons or ladles, and glasses, which were equated with the standard measures. For food items such as *roti*, *dosa*, *idli* and *vada*, the dimensions and numbers were recorded.

Procurement of ingredients: Finger millet, little millet and pearl millet were procured from wholesale markets. Other ingredients such as legumes, vegetables, oil, spices and salt were purchased from local markets.

Selection of millets for recipe formulation: For the recipe formulation, millet varieties with high nutrients were chosen. For example, pearl millet variety Dhanashakti was chosen for its high iron content and amino acids which can complement legumes to make a complete amino acid profile [21]. Finger millet, regardless of variety, contains three times the calcium in milk. Finger millet and pearl millet supply 50–100% of the daily value (DV) of methionine amino acid for children [22]. When mixed with legumes such as pigeonpea, these millets provide a complete amino acid profile [22] and, when combined with vegetables, they provide some major micronutrients. These millets were therefore introduced in the form of common south Indian food items, namely, *idli*, *khichdi*, *upma* and *bisibella bath*, in which rice was replaced by millets. The ingredients were selected considering their nutritional value and the added advantage of using them to enhance the absorption of nutrients. For example, to enhance iron absorption, the vitamin C content of the food was ensured by adding ingredients, such as capsicum. We also considered the feasibility of cooking each of the recipes in a large cauldron where the ingredients are untouched by humans right from the stage when the ingredients are added until the filling of the thermal container (fully automated) which was then carried in a warmer container to the schools through the established distribution system. 

Nutrient analysis: Food consumed by the sample children was classified into meat, eggs, cereals, pulses, green leafy vegetables, other vegetables, roots and tubers, milk and dairy products, fats and oils, and sugars. Nutrients consumed were classified into energy, protein, vitamins, and minerals [23]. The daily food and nutrient intake of the children was estimated using published food composition tables for Indian foods [19,20]. The average food and nutrient intake (for each item) was compared with the recommended dietary allowances (RDA) for adequacy stipulated by the Indian Council of Medical Research [24].

The formulated recipes were cooked, and both raw and cooked forms of food were analysed for targeted nutrients including carbohydrates, protein, fat, calcium, iron, zinc, and magnesium by AOAC method 19th edition [25]. The nutrient values per serving were then calculated based on the average daily intake by the sample children. 

Sensory evaluation: Sensory evaluation of aroma, appearance/colour and taste was conducted among the sample children to determine the acceptability of each recipe [26]. The five-point hedonic scale was adopted (1 being highly disliked and 5 being highly liked). For the children to express their degree of like or dislike, relevant emoji pictures were used, and they were asked to score the sensory attributes. The recipe was adjusted and finalized on the basis of the sensory evaluation and nutritional values.

Anthropometric measurements: The height and weight of the sample children from both the intervention and control groups were measured at the baseline and end line. The height, weight, and age of each child were used in the calculation of the height for age Z-score (HAZ), which is an indicator of stunting, and body mass index Z-score (BMIZ) which is an indicator of underweight, using the Epi Info 7 software (Centre for Disease Control, Atlanta, GA, USA) [27]. Based on the Z-scores, the children were categorized as severely undernourished, moderately undernourished, normal, and over-nourished when the Z-scores were below −3, between −3 and −2, between −2 and +2, and above +2, respectively [28,29]. 

Dietary intervention: The dietary intervention program was conducted for three months in both intervention schools. The meal was provided during mid-day in both intervention schools. The cauldron-cooked food from the Akshaya Patra kitchen were distributed in thermal containers to these two schools every day. To impart good practice on personal hygiene and to avoid food wastage, teachers and nutrition experts conducted sessions on hygiene and the importance of millet nutrients after the sensory evaluation and during the feeding program in focus groups and using interactive posters. The children were monitored by teachers to avoid food wastage and also wash their hands before eating. The average intake and wastage of food was estimated. Children were regularly encouraged to give feedback on the millet-based food. The feedback was used to adjust the recipe throughout the study period in terms of saltiness, spice and cooking qualities.

Data analysis: Descriptive statistics were used to present the nutritional values, participants’ profiles, sensory evaluation scores, undernutrition indicators, and costs per meal, while analysis of variance was conducted to determine the day to day variation in dietary intake obtained from the diet diary, and the paired *t* test [30] was employed to examine the statistical significance of changes in undernutrition indicators before and after the intervention program. 

More specifically, the acceptance of the recipes was determined by the mean scores out of 5.0, where the score of 3.0 represented neutral. That is, when the mean score was nearly 4.0, it is interpreted that the recipe was moderately liked. Likewise, a mean score nearly 5.0 implied the recipe being highly liked. As for the paired *t* test on Z-scores for undernutrition, the confidence level of 99% was applied, allowing only 1% occurrence of false identification of significant changes. Furthermore, since the expected direction of change was definite (positive), the one-tailed *p*-value was adopted for rejection of the null hypothesis of no change in the indicators. The statistical analysis was performed using Stata 15 (StataCorp, Texas City, TX, USA).

In addition to the recipe acceptance evaluation, cost analysis was performed to reinforce the assessment of the suitability of the recipe formulations. 

## 3. Results

Table 1 shows the basic profile of the households from which the children participated in our study. The average household size was about five persons in both the intervention and control groups. The house ownership rate was slightly higher in the intervention group. All the households in the study were headed by men and the majority of them were engaged in casual labour such as for construction, showing a negligible difference in proportion between the two groups. The studied children depended on mid-day meals since 38% and 49% of the children in the intervention and control groups, respectively, went to school without eating breakfast. 

Table 2 shows the gender composition of the children who participated in the study. In total, 56% of the participants were girls. The share of girls was slightly higher in the control group (63.6%) compared to the intervention group (46.4%).

The household dietary assessment indicated that fortified rice was a major staple occupying 70% of the weight of their diets, where it was eaten in different forms including blended rice (steam cooked or toasted) and boiled rice with or without lemon juice, tamarind juice, or tomatoes. The remaining 30% comprised of watery pigeonpea stew as well as vegetables. 

The baseline data showed that only 56% of the sample school children had breakfast everyday (Table 3), and 16% of them had breakfast five days a week. The rest had breakfast four days a week or less frequently.

The baseline data showed that the meals consumed by household members provided 50 to 60% of required calories, 50% of protein, 40 to 50% of iron, 60% of calcium, and 40–50% of zinc, based on RDA (Table 4). The majority of calories came from rice, protein from pigeonpea and other pulses like chickpea and green gram, calcium from finger millet and legumes, and iron from vegetables.

The diet diary confirmed that the adolescent children consumed rice-based lunch at school and the remaining two meals at home. Although the average number of food groups consumed was four, the consumption of vegetables and milk products was neither regular nor in adequate quantities. As a result, the nutrient intake of the sample children in both control and intervention group was found to be low compared to the RDA for that age group (Table 5). In particular, iron and calcium intake was far from the RDA. This is because only 10% of the children consumed finger millet though it was regularly consumed by the parents or adults at home, and all the children consumed milk provided by the school once a day; thus, there was no other way they received enough calcium.

The sensory evaluation of recipes revealed that in general the sample children had a positive perception of all the formulated recipes. The mean overall acceptability score ranged from 4.5/5 to 4.7/5, depending on the recipe (Table 6). Finger millet *idli*, *bisi belle bath* and *upma* were equally preferred recipes. There was no wastage of food during the intervention period, and the average per capita food intake during the mid-day meals was estimated to be 350 g for millet-based meals and 200 g for sambar (pigeonpea soup) which is mainly served with fortified rice in regular MDM or with finger millet *idli* in the current study. 

**Figure 2:**
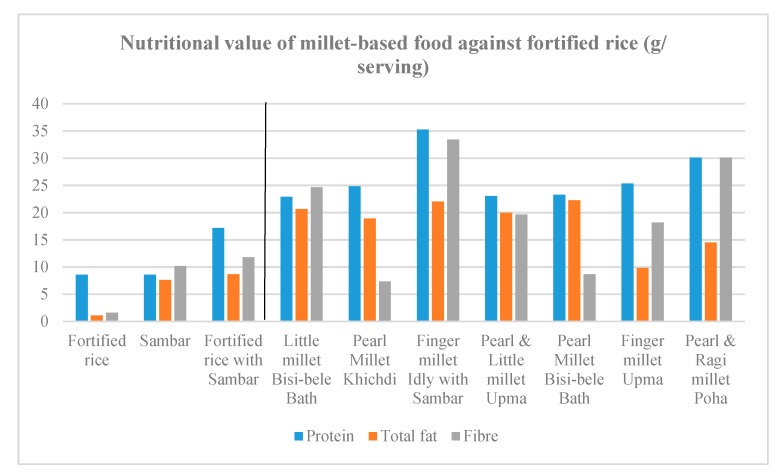
Protein, unsaturated fat and fibre content in millet-based meals in comparison with a fortified rice-based meal.

**Figure 3:**
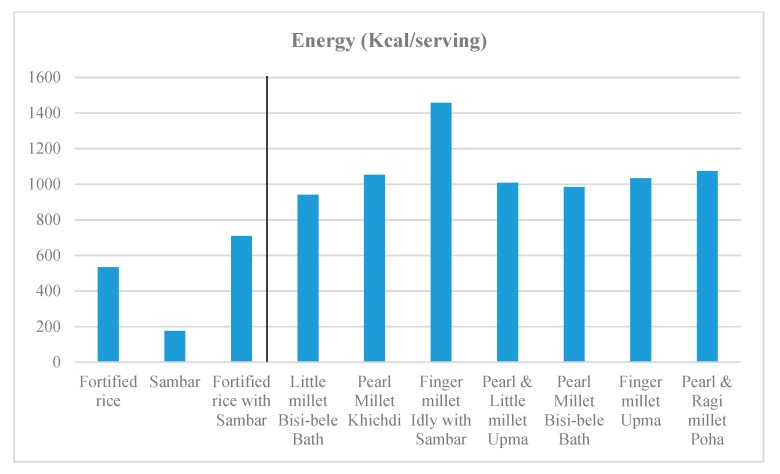
Energy content of the millet-based meals in comparison with a fortified rice-based meal.

**Figure 4:**
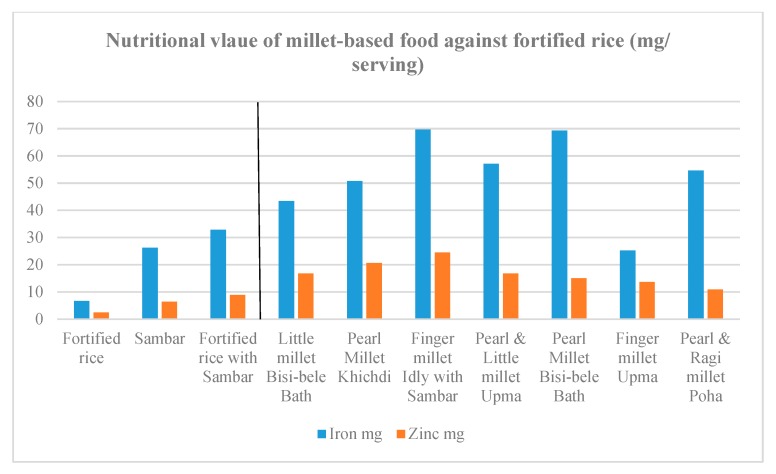
Iron and zinc content in the millet-based meals in comparison with a fortified rice-based meal.

**Figure 5:**
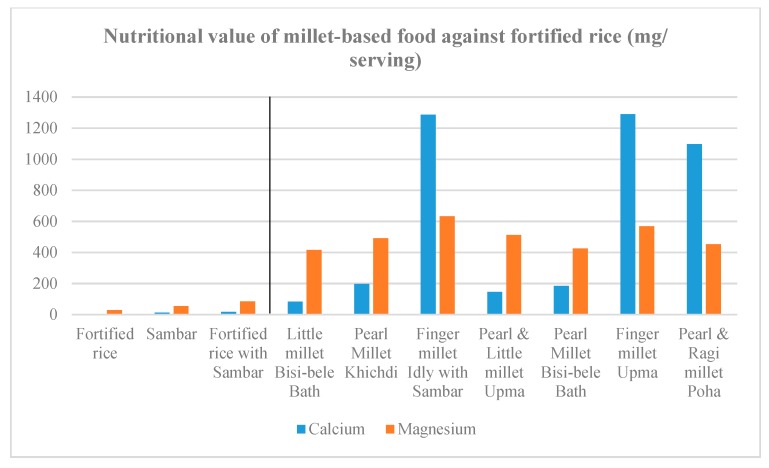
Calcium and magnesium content in the millet-based meal in comparison with a fortified rice-based meal.

Recipes were formulated to contain 60–100% millets. Other than millets, the recipes contained one or more legumes, such as dehulled green gram, pigeonpea and groundnut. The recipes also contained vegetables such as carrot, capsicum, and green peas. In terms of nutrient values, the recipes prepared with finger millet were confirmed to be high in calcium, iron, zinc and magnesium, and sufficient in energy and the type of protein complementary with the protein in pigeonpea. The recipes made from little millet and pearl millet were also high in iron, zinc, magnesium, energy and protein (Figure 2, Figure 3, Figure 4 and Figure 5). 

Table 7 presents the Z-scores for stunting and BMI at the baseline and end line for the two groups. On average, the sample children in both groups were within the “normal” range of undernourishment at both the baseline and end line, even though the average values were largely below the population average of zero. Between the two groups, the children in the intervention group were slightly more undernourished on average, which is presumably due to the non-random attrition from the original sample of 400 children. 

Lastly, and most importantly, Table 8 shows the one-tailed paired t test of the differences in Z-scores between the baseline and the end line. The result indicates that the changes were positive and statistically significant in the intervention group, and insignificant in the control group. The three-month MDM program with millet-based recipes resulted in raising the mean Z-scores for stunting and BMI by 0.07 and 0.166, respectively, compared to the control group. 

## 4. Discussion

The baseline result suggests that the children largely depended on mid-day meals for their calorie intake and other nutrient requirements. A similar trend was seen in children who received mid-day meals in Gujarat state [6]. 

The nutrient analysis of the millets-based meals formulated in this study shows that they are superior to fortified rice-based meals in nutritional value, which is mainly due to the combination of millets, legumes, and vegetables, providing balanced micro and macronutrients to the meal. As a result, a significant reduction in the extent of undernutrition was observed in this study.

The preference for and acceptability of the formulated recipes were relatively high, which was mainly due to the cultural suitability of the preparations. For example, when the children were asked why they liked finger millet *idli*, they responded that finger millet at home was cooked in the form of rice-finger millet balls, and that it resembled the taste. In other words, the formulated food merely replaced or supplemented the rice component in the conventional recipes cooked in Karnataka. The baseline also showed that all the children were already familiar with finger millet (Table 3) included in their food. It may be one of the reasons why their baseline RDA for calcium reached 50–60%. The intake of other nutrients by the children did not meet RDA standards (Table 5), which is in consonance with other studies conducted in India [31,32]. 

Pearl millet-based meals had high acceptability. Although an assessment of iron intake among the children was beyond the scope of this study, [33] found that intake of high iron pearl millet-based meals could reduce iron deficiency anaemia. Given the widespread iron deficiency anaemia among school going children in India, providing pearl millet meals may be a feasible way of reducing dietary iron deficiency anaemia [34]. 

However, it must be noted that several studies show that millets, as well as many other cereals and legumes, contain antinutrients, such as phytic acid, which affects the micronutrient absorption [12]. Other studies show that pearl millet exhibits goitrogenic effects [13,35,36]. Various processing and cooking methods have been suggested to mitigate the level of antinutrient contents in millets [12]. Furthermore, a recent study on breeding of pearl millet analysed the content of phytic acid and goitrogenic C-glycosylflavones (C-GFs) in pearl millet grains, providing information on genes involved in their biosynthesis. It was shown that large variability existed in both phytic acid and C-GFs contents, implying immense potential for selecting germplasms that are low in these contents, which would help maximize the benefits out of nutrients present in millet-based food [37]. In the current study, pearl millet was included in MDM twice a week and there was no record of developing mineral deficiency or thyroid related problems with the study sample. Yet, we suggest that care be taken when designing millet-based dietary interventions, particularly for children, and that appropriate preparation and cooking methods be imparted in consultation with culinary experts to mitigate the risk.

The children’s involvement in training sessions was intense with periodical feedback on the cooking quality of the millet-based meals. This helped the chefs adjust the meals according to the tastes and requirements of the children. The knowledge on nutritional benefits and interest in consumption improved, which could be one of the reasons why the children consumed the millet-based meals regularly for three months and never wasted any portion of their meal. A similar study conducted in Malawi shows that intensive training for mothers on diversified nutrition, food safety and hygiene improved the knowledge and nutritional outcome of undernourished children in three weeks’ time [38]. 

Table 9 shows the costs of preparing the different types of meals discussed suggesting that there is currently a cost implication in disseminating millet-based MDM programs. The cost per meal for the recipes based on little millet, pearl millet, and finger millet was calculated to be 50%, 37% and 21% higher, respectively, compared to that of conventional meals based on rice. However, if the rice-based meals were supplemented by additional ingredients to the levels of nutrition equivalent to the millet-based meals, the cost disadvantage would cancel out. The cost per millet-based meal was calculated based on the open market prices of the ingredients. If millet grains were subsidized as rice is for MDM, then the cost per meal would decrease tremendously. The Department of Food and Public Distribution allocates rice and wheat to various states and Union Territories at National Food Security Act (NFSA) prices, i.e., INR 2 per kg (USD 0.029) of rice and INR 3 per kg (USD 0.043) of wheat [39] under the national program of MDM. In Karnataka, the Public Distribution System (PDS) started distributing finger millet and sorghum in 2014. The cost of millets differs across various markets. For example, the farm gate price millet is approximately 50% less than the market price, but through government procurement, the farmers receive 20–30% higher price than the MSP for finger millet and sorghum compared to open market prices. Hence, selling farm produce through public procurement scheme or regulated market would inevitably be the choice for farmers to sell their produce as long as other issues such as late payment and procurement time periods are addressed. Considering the current consumption of finger millet in parts of rural and urban Karnataka, the government is able to procure 23% of the produced millets to distribute at the rate of 5 kg/household/month [40]. This might still not be sufficient if millets are introduced in school feeding programs. Successful provision would require strengthened value chains of these Smart Food crops, which are stress-resistant, nutrition dense, and environmentally sustainable. 

Potential for additional millet consumption through MDM: Over the last two decades, the millet consumption in India decreased from 12 kg to 9 kg per capita [41] mainly due to the availability of subsidized rice and wheat through PDS, increased income, urbanization and changing tastes and preferences of the consumers. However, with the introduction of good millet-based recipes preferred by school children, the consumption of highly nutritious millets would increase. Using India’s largest mid-day meal provider, Akshaya Patra as an example, Table 10 provides the information on additional millet requirements under different feeding frequency scenarios in Karnataka and in 12 other states of India. For Karnataka alone, feeding 480,000 school children with millet-based recipes would require around 17,690, 5054 and 2527 metric tons (MT) of millets annually for feeding daily, twice and once a week, respectively. 

Feeding 1.78 million children in 12 states of India would require about 39,166, 11,940 and 5595 MT of millets annually for feeding daily, twice and once a week, respectively. This additional demand for millets would encourage farmers to increase areas planted to these nutritious and climate resilient Smart Food crops.

Nonetheless, MDM monitoring and evaluation reports indicate that mid-day meals in India are largely made of major cereals (rice and wheat), with small amounts of vegetables and legumes because of their high prices [9]. Since millets are nutritious, complementary to legumes, and are cheaper when supplied through the PDS, millet-based recipes could provide the much needed balanced nutrition at a lower cost compared to adding vegetables and legumes to the rice-based meals. Furthermore, the additional cost in millet-based meals needs to be compared with the additional benefit arising from the nutritional outcome. This requires further research on the long-term impacts, including potential positive externalities to wider societies. 

Strengths and limitations of the study: To our knowledge, this study gave the first controlled behavioural intervention to assess the impacts of school meal interventions on the undernutrition indicators among children in India. Nonetheless, there are mainly four limitations in this study. First, the MDM dietary intervention provided in the school only covered estimated 40–45% of the children’s daily energy intake and, hence, the exact attribution to children’s growth would be difficult. The study focused on providing a proof-of-concept by presenting evidence of the effect of consuming millet-based food recipes in school MDM on children’s physical growth, rather than changing the full diets of the children. Since nearly a half of the studied children skipped breakfast, they consumed about 17.5 meals (2.5 × 7) per week on average, out of which five meals received intervention (i.e., 29% of all meals). With complete changes in dietary habits, the positive impact may double or triple, assuming linear relations. Second, our sampling framework included the purposive sampling element where only children meeting certain criteria of good practices were selected into the sample. This suggests that the positive effects found in this study may not represent children who are possibly less advantaged than the sample children. If such segments were included into the study, the magnitude of the positive effects may increase, though the exact quantification is left to further research. Third, the study did not measure the impact of millets based meals on biochemical parameters such as blood haemoglobin, which would potentially deepen the understanding of the channels of the observed positive impacts. Fourth, the study did not monitor the iodine status of the children and recommends a long term study to monitor the iodine status using different varieties of pearl millet.

## 5. Conclusions

This study, undertaken in the peri-urban area in Karnataka state of India, demonstrated three important pieces of evidence: (1) that the introduction of millet-based meals in school feeding programs can significantly improve the nutritional outcome of school going children compared to fortified rice-based meals; (2) that these meals can be enjoyed by the children; (3) that it can be cost effective if millets are given government pricing support as equally as rice. It is notable that the nutritional benefits were positive in just a three-month period when other factors such as the health status of the children was normal; and that these positive improvements were in comparison to fortified meals. Additionally, of consequence is that the acceptability of the millet-based meals was high, which is probably due to the efforts taken in designing the menu items in a culturally sensitive way. The efficacy of adding millets to the meals was backed by science which provided the foundation and clarity for ensuring that acceptability and nutritional benefits were realized.

The following key lessons were learnt on introducing millets in school meals: the importance of a recipe design that is culturally sensitive and suitable to the cooking equipment/process; the value of selecting both ‘type’ of millets to target specific nutrient needs and ‘variety’ of millets to significantly increase the nutrient levels; the importance of using all ingredients (including legumes and vegetables) in designing the menu to ensure a balanced meal, and selecting the right cooking methods and food combinations to maximize bioavailability (absorption of the micronutrients); how hygiene and food safety need to be maintained to ensure bioavailability of nutrients; and the significance of edutainment in influencing children and teachers in accepting the meals. As the participants were school-going children from a southern state of India, generalizability of the findings may be restricted to adolescent populations in parts of South Asia where dietary habits and culinary culture have commonalities.

The study recommends future research and development initiatives such as clinical testing to clarify the nutritional and health benefits; applied studies of other geographic/cultural areas that have different taste preferences to build up suitable recipes; and testing the effectiveness of different approaches to edutainment programs in schools around nutrition and millets to build knowledge and a positive modern image around millets. It is also recommended that to maximize the benefits of introducing millets into MDMs, the government include high micronutrient millets in the minimum support price scheme; connect farmers directly to the supply chains for MDMs; and ensure the supply of specific varieties of high iron millets.

## Figures and Tables

**Table 1:** Socioeconomic and demographic profile of households of the sample children.

Socio Economic and Demographic Profile	Unit	Intervention Group *n* = 160	Control Group *n* = 160
Household size (average)	Headcount	5.0	5.3
Type of house			
Rental	%	68.7	80.0
Owned	%	31.3	20.0
Sex of the household (HH) head			
Men	%	100	100
Women	%	0	0
Main occupation of HH head			
Employee	%	12.5	16.8
Self employed	%	11.8	8.7
Casual labor	%	62.5	65.0
Farming	%	0.0	0.0
No fixed jobs	%	13.1	9.3
Eating habits			
Children go to school without having any breakfast (Yes/No)	% of Yes	38.2	48.8
Children eat lunch at school (Yes/No)	% of Yes	100	100
Households with rice as staple (Yes/No)	% of Yes	100	100

**Table 2:** Gender balance of the sampled children.

	Control	Intervention	Aggregate
Boys (%)	36.4	50.7	44.4
Girls (%)	63.6	49.3	55.6
Total (%)	100	100	100
Number of children who participated	107	136	243

**Table 3:** Food habit related questions and children’s response during the baseline (a few selected example questions).

Baseline Questions Asked (Selected Examples)	Response	Number of Children (*n* = 320)	Percentage
1. How frequently do you take breakfast?			
	Everyday	181	56.6%
	5 days a week	53	16.6%
	2 to 4 days a week	49	15.3%
	Rarely	12	3.8%
	Not at all	22	6.9%
	Not sure	3	0.9%
2. Which millet is commonly used at your home?			
	Finger millet	320	100%
	Finger millet, Sorghum and pearl millet	58	18.1%
3. How do you describe your dietary habit?			
	Vegetarian (Not vegan)	21	6.5%
	Both vegetarian with occasional non-vegetarian	300	93.5%

**Table 4:** Estimated average per capita macro and important micronutrient intake of the based on household dietary assessment in comparison to RDA for adults and adolescents.

Nutrients	Amount of Nutrient from Current Food Average (SD)	RDA for Adults * (Moderately Active)		RDA for Adolescent * (10–14 years)	
		Men (60 kg)	Women (55 kg)	Girl	Boy
Energy (Kcal)	1469 (211)	2730	2230	2010–2330	2190–2750
Protein (g)	28.72 (6.74)	60	55	36.8–49.0	36.3–43.3
Fat (g)	26.38 (12.77)	30	25	35–40	35–45
Iron (mg)	9.23 (3.17)	17	21	21–32	27
Calcium (mg)	441.8 (213.8)	750	750	800	800
Zinc (mg)	6.02 (1.55)	12	10	9–11	9–11

Source: Authors’ survey; * RDA is based on Recommended dietary allowance for Indians [24].

**Table 5:** Estimated nutrient intake recorded in three-day diet diary compared to the recommended dietary allowance (RDA) for adolescent girls and boys.

Nutrients	Average Intake (SD)		* RDA for Adolescent (10 to 15 years)									
	Girls	Boys	Girls					Boys				
	10–15 Years	10–15 Years	10–11 Years	11–12 Years	12–13 Years	13–14 Years	14–15 Years	10–11 Years	11–12 Years	12–13 Years	13–14 Years	14–15 Years
Energy (Kcal)	1681 (275)	1516 (366)	2010	2010	2330	2330	2330	2190	2190	2750	2750	2750
Protein (g)	36.04 (4.41)	32.03 (5.88)	36.8	40.0	44.5	49.0	52.8	36.3	39.6	43.7	49.8	54.7
Fat (g)	8.96 (0.55)	7.70 (0.74)	35	35	40	40	40	35	35	45	45	45
Iron (mg)	20.71 (3.42)	18.68 (4.56)	27	27	27	27	27	21	21	32	32	32
Calcium (mg)	334.61 (3.42)	285.50 (3.36)	800	800	800	800	800	800	800	800	800	800
Zinc (mg)	8.22 (1.26)	7.39 (1.68)	9	9	11	11	11	9	9	11	11	11

Source: Authors’ survey; * RDA is based on Recommended dietary allowance for Indians [24].

**Table 6:** Preference and acceptability of recipes based on sensory attributes.

Name of the Recipe	Taste Mean (SD)	Appearance Mean (SD)	Smell Mean (SD)	Overall Acceptability Mean (SD)	Rank Based on Overall Acceptability
Little millet bisi belle bath	4.8 (0.6)	4.4 (0.8)	4.5 (0.9)	4.7 (0.7)	1
Pearl millet Kitchadi	4.5 (0.9)	4.3 (1.0)	4.4 (1.0)	4.5 (1.0)	5
Finger millet idli with Sambar	4.7 (0.8)	4.5 (0.9)	4.6 (0.7)	4.7 (0.8)	2
Little millet rice with Rasam	4.5 (1.1)	4.4 (0.9)	4.5 (0.9)	4.6 (0.9)	4
Upma	4.6 (0.9)	4.5 (0.8)	4.6 (0.8)	4.7 (0.8)	2

**Table 7:** Undernutrition indicators for the sampled children at baseline and end line by treatment status.

	Height for Age Z-Score		BMI for Age Z-Score	
	Baseline	End Line	Baseline	End Line
Treated (*n* = 136)	−1.548 (1.247)	−1.474 (1.269)	−1.487 (1.550)	−1.321 (1.529)
Control (*n* = 107)	−1.207 (1.027)	−1.163 (1.007)	−1.002 (1.250)	−1.006 (1.265)

NB: Means are presented and standard deviations are in parentheses. The end line is three months after the baseline.

**Table 8:** Paired t test of the change in mean Z-scores for undernutrition indicators from the baseline to the end line by treatment status.

	Height for Age Z-Score			BMI for Age Z-Score		
	Δ [End line–Baseline]	*t*-Statistic	*p*-Value	Δ [End Line–Baseline]	*t*-Statistic	*p*-Value
Treated (*n* =136)	0.074	5.204	0.000	0.166	2.817	0.003
Control (*n* = 107)	0.044	0.3842	0.351	−0.004	−0.028	0.511

NB: Δ indicates the change in mean. One-tailed *p*-values are presented. The end line was three months after the baseline.

**Table 9:** Cost per meal for millet-based meal compared to rice in MDM, 2018.

Cost Per Meal for Millet-Based Meals Compared to Rice Meals in MDM *		
Type of Meal in MDM	Cost Per Meal (INR)	% Increase in Cost for Millet-Based Meal Compared to Rice-Based Meal
Rice-based meal **	11.4	NA ***
Little millet-based meal	22.7	50
Pearl millet-based meal	18.2	37
Finger millet-based meal	14.6	21

* The cost is calculated based on market prices in Karnataka and is not applicable to all the other states. ** Rice is subsidized by the government. *** NA = Not applicable.

**Table 10:** Required volume of millets for MDM programs under three different feeding frequency scenarios.

Region	Number of Children	Feeding Scenarios		
		Daily	Twice a Week	Once a Week
Karnataka state	486,172	340.2 tonnes/week	97.2 tonnes	48.6 tonnes
		17,690 tonnes/annum (340.2 tonnes × 52 weeks)	5054.0 tonnes/annum (97.2 × 52 weeks)	2527.2 tonnes/annum (48.6 × 52 weeks)
12 states of India	1.76 million	741.2 tonnes/week	215.2 tonnes/week	107.6 tonnes/week
		39,166 tonnes/annum (741.2 tonnes × 52 weeks)	11,190.4 tonnes/annum (215.2 tonnes × 52 weeks)	5595.2 tonnes/annum (107.6 tonnes × 52 weeks)
